# Supplementary material for: Short-Term Consumption of Cuban Policosanol Lowers Aortic and Peripheral Blood Pressure and Ameliorates Serum Lipid Parameters in Healthy Korean Participants: Randomized, Double-Blinded, and Placebo-Controlled Study
Source: Int J Environ Res Public Health. 2019 Mar 5;16(5):809. doi: 10.3390/ijerph16050809 (PMC6427682; doi:10.3390/ijerph16050809)
Supplement: Supplementary file 1 [file ijerph-16-00809-s001.pdf]

Supplementary material

Table S1. Pearson's correlation analysis between at the baseline and after 12 weeks in the placebo group.

Variables		TC	TG	HDL	% HDL	TG/HDL	LDL	LDL/HDL	MAP
**0 week**									
**Peripheral**	SBP	0.262	−0.056	−0.154	−0.272	−0.030	−0.296	0.281	0.889 *
	DBP	0.357	0.245	−0.212	−0.437*	0.212	0.340	0.354	0.955 *
**Aortic**	SBP	0.457 **	−0.127	−0.059	−0.313	−0.115	0.485 **	0.388	0.822 *
	DBP	0.099	0.163	−0.121	−0.145	0.149	0.084	0.136	0.877 *
**12 weeks**									
**Peripheral**	SBP	−0.078	0.020	0.022	0.110	−0.050	−0.097	−0.093	0.928 *
	DBP	−0.118	0.009	−0.053	0.050	−0.046	−0.124	−0.077	0.978 *
**Aortic**	SBP	−0.137	−0.046	0.033	0.131	−0.100	−0.154	−0.167	0.812 *
	DBP	−0.136	0.005	0.062	0.145	−0.064	−0.169	−0.182	0.898 *

SBP, systolic blood pressure (mmHg); DBP, diastolic blood pressure (mmHg); TC, total cholesterol; TG, triglyceride (mg/dL); HDL-C, high density lipoprotein cholesterol (mg/dL); % HDL, percentage of high density lipoprotein cholesterol; TG/HDL-C, triglyceride ratio high density lipoprotein cholesterol; LDL, low density lipoprotein cholesterol (mg/dL); LDL/HDL; LDL ratio high density lipoprotein cholesterol; MAP, mean arterial pressure. * Correlation is significant at the 0.01 level (2-tailed). ** Correlation is significant at the 0.05 level (2-tailed).

Table S2. Pearson's correlation analysis at the baseline and after 12 weeks of consuming policosanol 10 mg.

Variables		TC	TG	HDL	% HDL	TG/HDL	LDL	LDL/HDL	MAP
**0 week**									
**Peripheral**	SBP	0.297	0.237	−0.119	−0.252	0.240	0.226	0.264	0.936 *
	DBP	0.270	0.274	−0.069	−0.204	0.262	0.165	0.199	0.968 *
**Aortic**	SBP	0.410 **	0.234	0.007	−0.265	0.221	0.320	0.257	0.905 *
	DBP	0.098	0.133	0.141	0.037	0.110	0.004	−0.072	0.766 *
**12 weeks**									
**Peripheral**	SBP	0.238	0.454 **	−0.255	−0.348	0.461 **	0.060	0.219	0.896 *
	DBP	0.248	0.373	−0.388	−0.456 **	0.401	0.148	0.315	0.956 *
**Aortic**	SBP	0.289	0.395	−0.215	−0.346	0.432 **	0.131	0.270	0.792 *
	DBP	0.220	0.126	−0.275	−0.374	0.145	0.221	0.293	0.837 *

SBP, systolic blood pressure (mmHg); DBP, diastolic blood pressure (mmHg); TC, total cholesterol; TG, triglyceride (mg/dL); HDL-C, high density lipoprotein cholesterol (mg/dL); % HDL, percentage of high density lipoprotein cholesterol; TG/HDL-C, triglyceride ratio high density lipoprotein cholesterol; LDL, low density lipoprotein cholesterol (mg/dL); LDL/HDL; LDL ratio high density lipoprotein cholesterol; MAP, mean arterial pressure. * Correlation is significant at the 0.01 level (2-tailed). ** Correlation is significant at the 0.05 level (2-tailed).

Table S3. Pearson's correlation analysis at the baseline and after 12 weeks of consuming policosanol 20 mg.

Variables		TC	TG	HDL	% HDL	TG/HDL	LDL	LDL/HDL	MAP
**0 week**									
**Peripheral**	SBP	0.039	0.343	−0.201	−0.142	0.327	−0.074	0.010	0.793 *
	DBP	0.381 **	0.516 *	−0.009	−0.190	0.454 **	0.173	0.093	0.967 *
**Aortic**	SBP	0.306	0.407 **	−0.214	−0.270	0.391 **	0.190	0.174	0.744 *
	DBP	0.374 **	0.490 *	−0.048	−0.212	0.439 **	0.187	0.122	0.861 *
**12 weeks**									
**Peripheral**	SBP	0.047	0.401 **	−0.121	−0.148	0.424 **	−0.107	−0.029	0.921 *
	DBP	0.267	0.371	−0.145	−0.366	0.410 *	0.129	0.184	0.975 *
**Aortic**	SBP	0.064	0.319	−0.072	−0.107	0.357	−0.065	−0.015	0.840 *
	DBP	0.202	0.317	−0.046	−0.213	0.343	0.065	0.082	0.835 *

SBP, systolic blood pressure (mmHg); DBP, diastolic blood pressure (mmHg); TC, total cholesterol; TG, triglyceride (mg/dL); HDL-C, high density lipoprotein cholesterol (mg/dL); % HDL, percentage of high density lipoprotein cholesterol; TG/HDL-C, triglyceride ratio high density lipoprotein cholesterol; LDL, low density lipoprotein cholesterol (mg/dL); LDL/HDL; LDL ratio high density lipoprotein cholesterol; MAP, mean arterial pressure. * Correlation is significant at the 0.01 level (2-tailed). ** Correlation is significant at the 0.05 level (2-tailed).
